# Fit of biokinetic data in molecular radiotherapy: a machine learning approach

**DOI:** 10.1186/s40658-024-00623-5

**Published:** 2024-02-22

**Authors:** Davide Ciucci, Bartolomeo Cassano, Salvatore Donatiello, Federica Martire, Antonio Napolitano, Claudia Polito, Elena Solfaroli Camillocci, Gianluca Cervino, Ludovica Pungitore, Claudio Altini, Maria Felicia Villani, Milena Pizzoferro, Maria Carmen Garganese, Vittorio Cannatà

**Affiliations:** 1 Medical Physics Unit, Bambino Gesù Children’s Hospital, IRCCS, Rome, Italy; 2 Tor Vergata Postgraduate School of Medical Physics, University of Rome, Rome, Italy; 3 Medical Physics Department, IRCCS Regina Elena National Cancer Institute, Rome, Italy; 4 Roma 3 University of Rome, Rome, Italy; 5 Nuclear Medicine Unit/Imaging Department, Bambino Gesù Children’s Hospital, IRCCS, Rome, Italy

**Keywords:** Machine learning, Akaike information criterion, F-test, Fit function, Biokinetic curves

## Abstract

**Background:**

In literature are reported different analytical methods (AM) to choose the proper fit model and to fit data of the time-activity curve (TAC). On the other hand, Machine Learning algorithms (ML) are increasingly used for both classification and regression tasks. The aim of this work was to investigate the possibility of employing ML both to classify the most appropriate fit model and to predict the area under the curve (τ).

**Methods:**

Two different ML systems have been developed for classifying the fit model and to predict the biokinetic parameters. The two systems were trained and tested with synthetic TACs simulating a whole-body Fraction Injected Activity for patients affected by metastatic Differentiated Thyroid Carcinoma, administered with [^131^I]I-NaI. Test performances, defined as classification accuracy (CA) and percentage difference between the actual and the estimated area under the curve (Δτ), were compared with those obtained using AM varying the number of points (N) of the TACs. A comparison between AM and ML were performed using data of 20 real patients.

**Results:**

As N varies, CA remains constant for ML (about 98%), while it improves for F-test (from 62 to 92%) and AICc (from 50 to 92%), as N increases. With AM, $\Delta \tau$ can reach down to − 67%, while using ML $\Delta \tau$ ranges within ± 25%. Using real TACs, there is a good agreement between τ obtained with ML system and AM.

**Conclusions:**

The employing of ML systems may be feasible, having both a better classification and a better estimation of biokinetic parameters.

## Background

Biokinetic parameters play a crucial role in molecular radiotherapy (MRT) in the assessment of the absorbed dose to lesions or organs at risk as they are closely related to treatment toxicity and efficacy [1].

Determining the time integrated activity ($\tau$) accurately is challenging [2, 3]. The assessment of Time Activity Curve (TAC) depends on the data (TAC-Ps) collection and on the choice of the model that best fits data, in order to calculate the time integrated activity coefficient (TIAc). Many parameters affect the fit model selection: the number of TAC-Ps, the time window, the time sampling and the error that affects each measurement.

In 2007 Glatting et al. [4] proposed the use of the corrected Akaike Information Criterion (AICc) and F-test [5–8] methods for the comparison of two different models in molecular radiotherapy. In 2013 Kletting et al. [9] showed the need of using a fitting method dedicated to MRT data whereby the physical and biological aspects of the biokinetics curve must be considered in order to compute meaningful parameters.

In recent years, several applications based on Machine Learning (ML) algorithms have been developed in nuclear medicine [10–12]. The first aim of this study was to implement ML systems to classify the proper curves model and predict, via regression, the TIAc. The secondary aim was to compare the ML performances with the ones obtained with fit algorithm and the analytical methods, AICc and F-test.

## Methods

In order to have a better understanding of this manuscript we assume the following definitions: (i) Points of the Time-Activity Curve (TAC-Ps) are the set of points used to determine a TAC (ii) The training set (Tr_N_) and the test set (Ts_N_) consist of 10,000 and 2000 TAC-Ps, respectively, where each individual TAC-Ps is composed of a number of points equal to N.

**Fig. 1:**
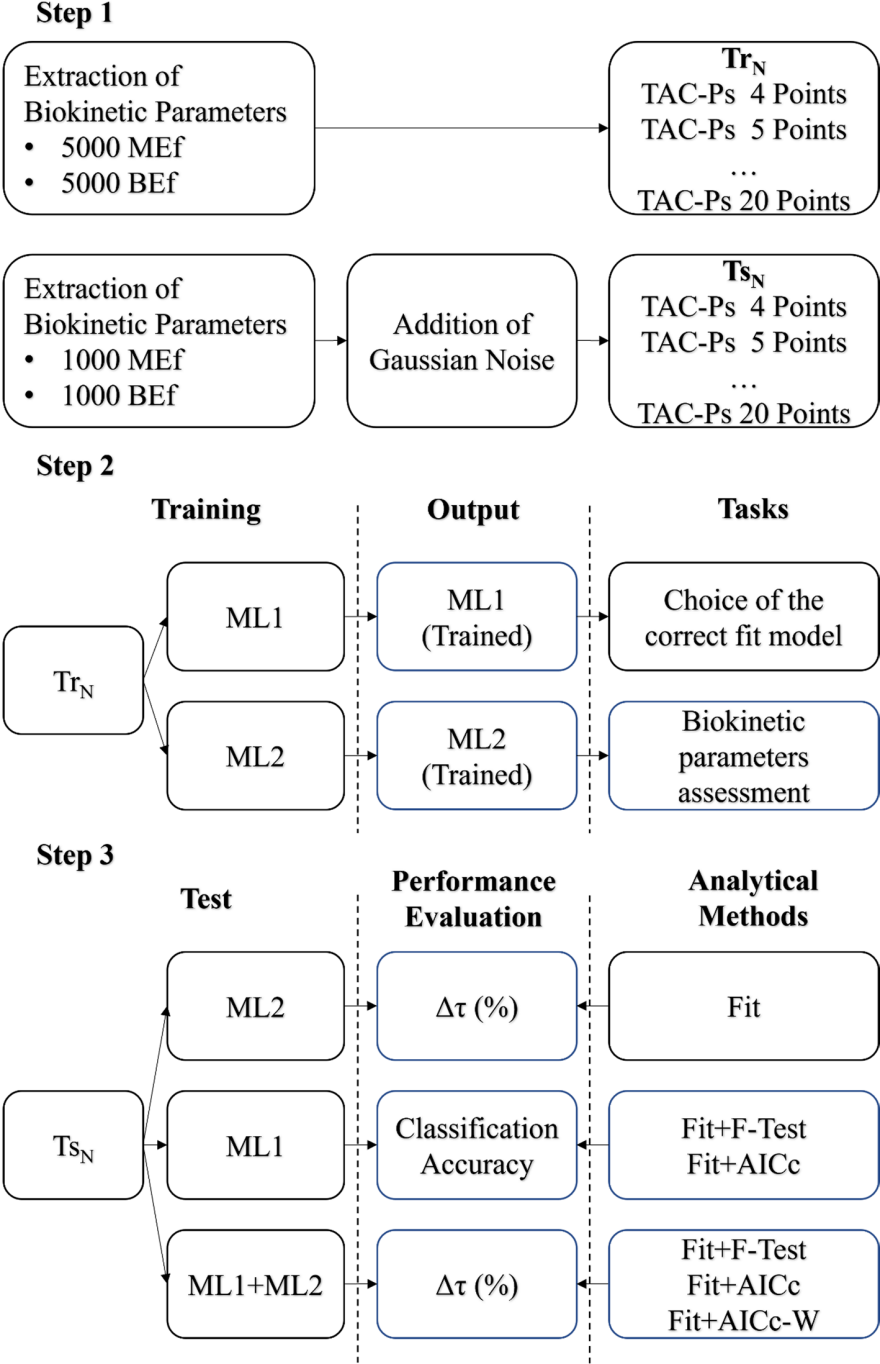
Study workflow

The study was subdivided in 3 steps: (i) the generation of synthetic Tr_N_ and Ts_N_ to train and test the machine learning systems. (ii) The training of the ML systems, (iii) the test of the ML systems and performance evaluation in comparison with analytical methods. All these steps are fully described in the sections below and shown in Fig. 1.

At the end, in order to show the applicability of the developed methods, the ML systems were used to calculate the TIAc of 20 patients’ TAC-Ps.

### Training and test dataset generation

To train and test the ML systems a series of Tr_N_ and Ts_N_ were synthetically generated. The aim of this first step was to generate TAC-Ps simulating whole-body fractions of injected activity (FIA_WB_) of patient affected by metastatic Differentiated Thyroid Carcinoma (mDTC), defined as:

1 $$FI{A}_{WB}\left(t\right)=\frac{{A}_{WB}\left(t\right)}{{A}_{Adm}}$$

where ${A}_{WB}\left(t\right)$ is the whole-body activity at time t and ${A}_{Adm}$ is the administered activity.

**Fig. 2:**
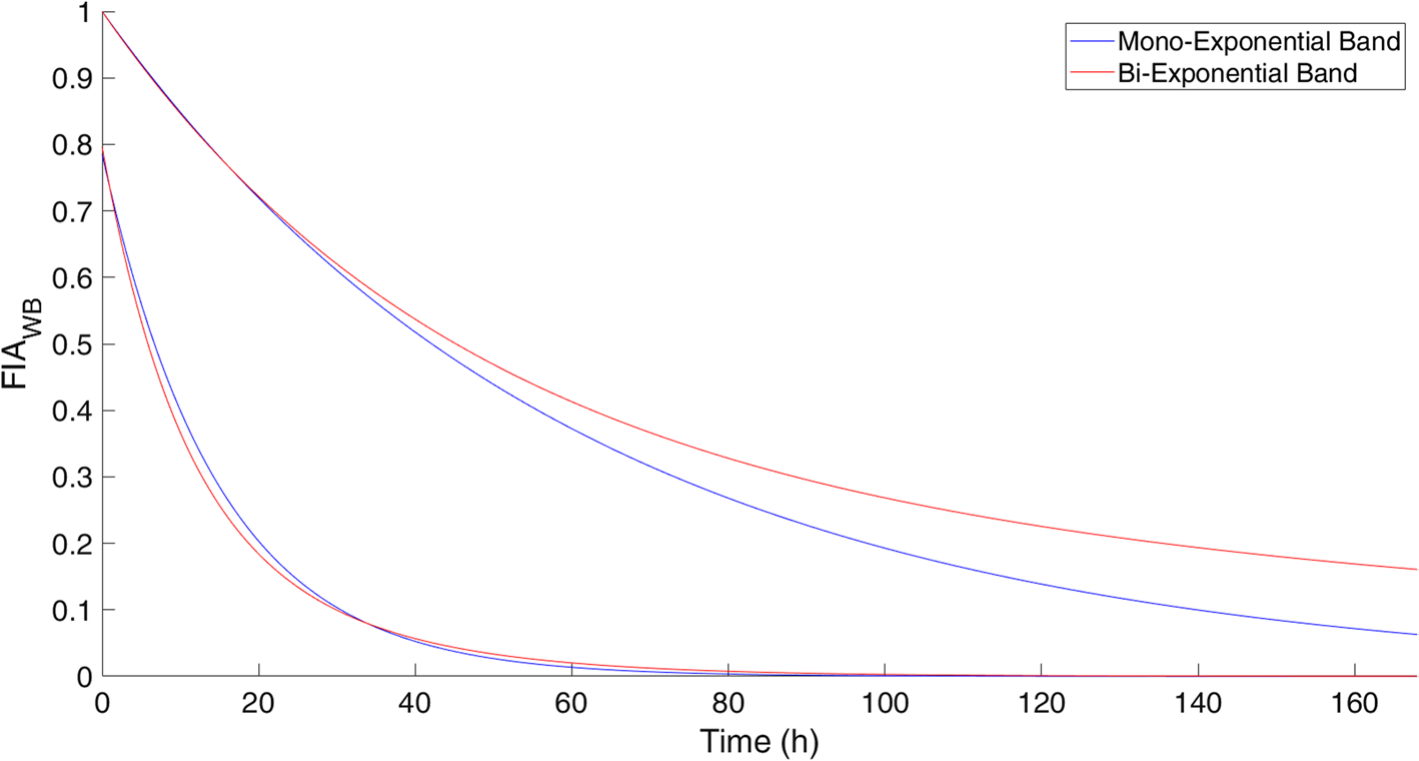
All the generated TACs are into the blue and red bands for Mono-Exponential and Bi-Exponential functions respectively

The synthetic TAC-Ps were generated with both a Mono-Exponential (MEf) and Bi-Exponential functions (BEf) into a band of possible curves, showed in Fig. 2, and their values are listed in Table 1. In the “Appendix” the generation method is reported.

To study how N affects the ML performances, 10 different Tr_N_ were created, with N ranging from 4 to 20, in a time window of 160 h. In order to simulate the patient’s hospitalization, the first N-1 points were equally distributed in a range of [0; 54] h, while the last point was set at 160 h. Each Tr_N_ was used to train the ML systems and it was derived from 5000 MEf and 5000 BEf curves.

Similarly, 10 Ts_N_ were generated to test the ML systems; they were derived from 1000 MEf and 1000 BEf curves. The generation procedure is similar to the one of the Tr_N,_ with the addition of Gaussian noise on the FIA coordinates, in order to simulate measuring errors. The amount of added noise is randomly extracted from a Gaussian distribution centred on the FIA coordinates, with a standard deviation of 5% on the FIA values [13].

### Implementation and training of ML system

Two different supervised learning systems were implemented using Python scripts: one for a binary classification task (ML1) and one for a regression task (ML2). Scikit-Learn library provided different machine learning models.

Hardware was composed of a personal computer having 10th generation Intel I7 CPU with 16 GB RAM.

ML1 is an ensemble of two Logistic Regression models, which used the Soft Voting method to predict the proper class [14]. The TAC-Ps were implemented as features (2N features considering x and y coordinates), and it returns MEf or BEf class as output.

ML2 is an AdaBoost ensemble composed of 5 Gradient Boosting Regressors (GBRs), working sequentially. Each GBR was designed as a chain of 1000 Decision Tree Regressors [14, 16]. The ML2 task is to predict the TIAcs (${A}_{i}, {\lambda }_{i}$), in order to calculate $\tau$. In ML2, TAC-Ps and fit model (MEf or BEf) were used as input features (2N + 1 features). Each Tr_N_ with a specific number of points individually trains ML2. Therefore, even though the ML2 algorithms are always the same, ML2 consists of 10 ML2_N_, depending on the number of points N. To simplify, the subscript N is omitted when referring to ML2 in the rest of this text.

The adopted models from Python Scikit-Learn library were customized with hyperparameters [17] reported in Tables 2 and 3, for the classifier and the regressor respectively. All the other ones that are not reported in tables were set with the default values proposed by the library.

**Table 1:** TIAc Parameters for the band of possible TACs

	MEf		BEf			
	A_0_	λ_0_ [h^−1^]	A_1_	λ_1_ [h^−1^]	A_2_	λ_2_ [h^−1^]
Lower limit	0.78	0.0676	0.46	0.1078	0.34	0.0476
Upper limit	1.00	0.0165	0.70	0.0225	0.30	0.0044

**Table 2:** Scikit-Learn hyperparameters used for the customization of ML1

Hyperparameter	1st Estimator	2nd Estimator
solver	“liblinear”	“sag”
max_iter	100 (default value)	10,000

**Table 3:** Scikit-Learn hyperparameters used for the customization of ML2

Hyperparameter	GBR	AdaBoost Regressor
loss	l s	Linear (default value)
n_estimators	1000	5
learning_rate	0.1 (default value)	0.1
max_depth	5	N.A

### Analytical methods: AICc and F-test

Each TAC-Ps of Ts_N_ were fitted using the Trust-Region algorithm, implemented on the Matlab toolkit, while a stripping algorithm was used to evaluate the fit starting point [18]. Each TAC-Ps was fitted both with MEf and BEf and the two results were compared using the AICc and F-Test (with a confidence interval of 95%), as mentioned by Kletting et al. [4].

As reported in [7] the AICc is described by the following equation:

2 $$AICc=N\cdot {\text{ln}}\left(\frac{SS}{N}\right)+2\cdot K+\frac{2\cdot K\cdot (K+1)}{N-K-1}$$

where K is the number of estimated parameters included in the model, N is the number of points, and SS is the sum of squared deviations (between the measurement and the fitted curve). The model with the lower AICc score is the model that is more likely to be correct. Once AICc has been calculated for each fitting model, it is possible to compute the probability that the correct model (i) has been chosen, as follows:

3 $${w}_{i}=\frac{{e}^{\left(-\frac{\Delta }{2}\right)}}{1+{e}^{-\frac{\Delta }{2}}}$$

where $\Delta$ is the difference between the AICc score.

The model with the minimal AICc value between all candidate models indicates the best model.

The F-test is a hypothesis test in which only two models can be compared using the following equation [4, 7]:

4 $$F=\frac{(S{S}_{ME}-S{S}_{BE})/S{S}_{BE}}{(D{F}_{ME}-D{F}_{BE})/D{F}_{BE}}$$

where $SS$ are the sums of squared deviations (for MEf and BEf) and DF are the degrees of freedom (DF = N-K). The decision to accept or discard the MEf model is based on the p-value calculated from the F ratio. For a P value below the chosen significance level (0.05) the MEf model is rejected and therefore the more complex model is assumed to fit the data in a significantly better way.

### Test and performance evaluation

A tenfold cross validation of ML1 system has been performed using 80% of the Tr_N_ in training and the remaining 20% for testing. The classification accuracy (CA) of the correct fit model was chosen as performance estimator. It is defined as:

5 $$CA=\frac{C{C}_{ME}+C{C}_{BE}}{2000}$$

where ${CC}_{ME}$ and $C{C}_{BE}$ are the number of correct classifications for mono- and bi-exponential functions respectively, and 2000 is the number of TACs.

The ability of ML1 system, AICc and F-test to correctly classify the model was evaluated, determining the CA using Ts_N_ as input.

The chosen parameter to evaluate the goodness of TIAc prediction was the area under each TAC ($\tau )$, assessed through the following equation [15]:

6 $$\tau =\sum_{i=1}^{n}\frac{{A}_{i}}{{\lambda }_{i}}$$

where $n$ is equal to 1 for MEf and 2 for BEf and ${A}_{i}$ and ${\lambda }_{i}$ are the parameters obtained from the fit.

Two different tests were performed to evaluate the goodness of TIAc prediction: the first had the aim to evaluate the best possible performances of the fit algorithm and ML2, considering a classification with no errors. Meanwhile the second test evaluated the performances of the chains ML1 + ML2, fit + AICc and fit + F-Test, considering all the classification errors. In addition, a fourth chain was considered, named fit + AICc-W. In this method, an average model was considered, taking into account the probability of each model given by the parameter ${w}_{i}$. The area $\tau$ was calculated as follows:

7 $$\tau ={\tau }_{MEf}\cdot {w}_{MEf}+{\tau }_{BEf}\cdot {w}_{BEf}$$

The performances of the two tests were evaluated assessing the distributions of the percentage differences $(\mathrm{\Delta \tau })$ between the calculated $\tau$ and true one, the interquartile range and the Maximum Error Range (MER). The MER was represented as the range between the minimum and maximum value of $\mathrm{\Delta \tau }$.

### Test on patient data

The whole-body TAC-Ps of 20 patients, affected by mDTC, were evaluated employing both methods: Fit + F-Test and ML1 + ML2. All TAC data were obtained by calculating the geometric mean of measurements taken with a plastic scintillator placed at a distance of 4 m from the patient and calibrated in terms of H*(10), providing a value of the dose rate in µSv/h.

Each TAC-Ps had 5 or 6 points and they were uniformly classified as 10 MEf and 10 BEf by the F-Test.

## Results

The computation time to train ML1 and ML2 sequentially is about eight minutes, while the computer takes few seconds to perform the test with 2000 curves. The same hardware takes about 10 min to perform fit + F-test and fit + AICc using the same datasets.

**Fig. 3:**
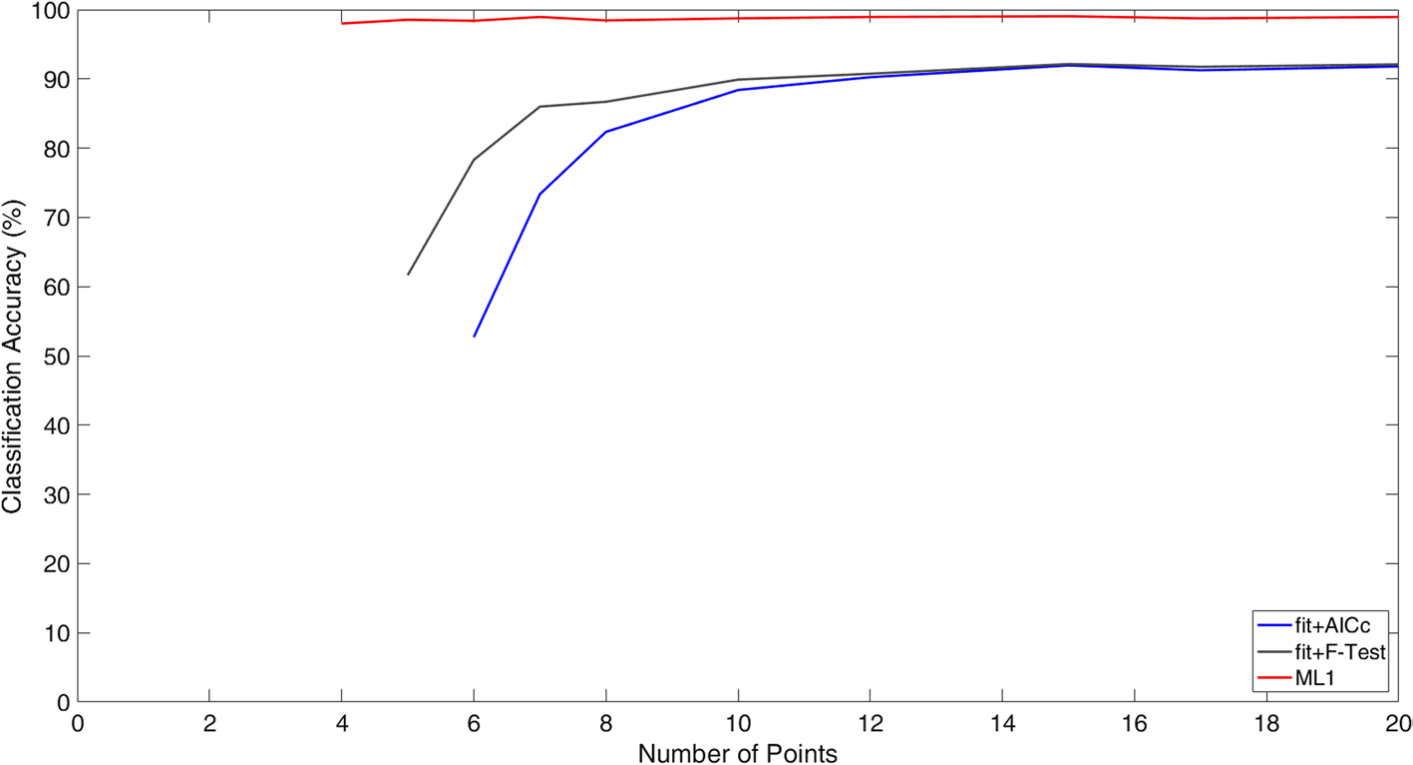
Classification accuracy obtained by three methods varying the number of points of the TACs simulated

The CA in the cross validation, varying the number of points (N), is reasonably constant at a value of 99%. Figure 3 shows the CA of the three systems using the test dataset that is about constant at the value of 98% for ML1, while it increases from 50 to 92% for AICc and from 62 to 92% for F-test, when the number of points increases.

**Fig. 4:**
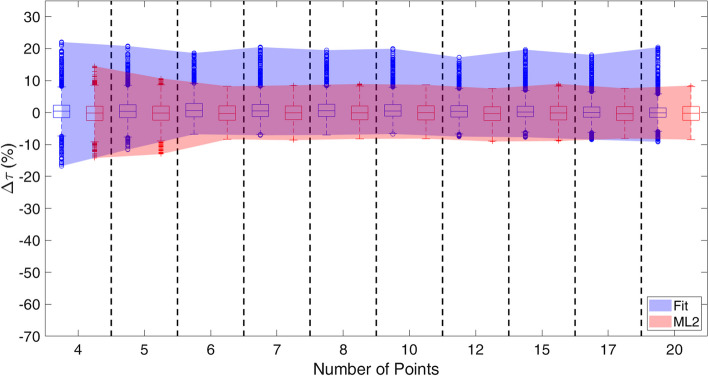
Boxplots representing the Δτ distributions and the MER bands obtained using the fit algorithm (blue) and ML2 (red). For each set of measures the performances of the ML system and the fit algorithm were compared only for the assessment of the AUC, since it was known a priori if the points follow a mono-exponential or a bi-exponential trend. Through the estimation of the fit model parameters the AUC was calculated and the distributions were represented

Figure 4 shows the $\mathrm{\Delta \tau }$ distributions obtained using a classification without errors. The median values remain approximately constant around 0% for both methods, and also the width of the distribution between the first and third interquartile is independent from the number of points with values equal to 4.5% and 3.6% for ML2 and the fit algorithm respectively. MER are represented by the bands in Fig. 4, which become thinner as the number of points increases, and their ranges vary from [− 14.2; 14.5]% to [− 8.1; 7.5]%, and from [− 16.8; 22.1]% to [− 6.7; 17.3]% for ML2 and fit algorithm respectively.

**Fig. 5:**
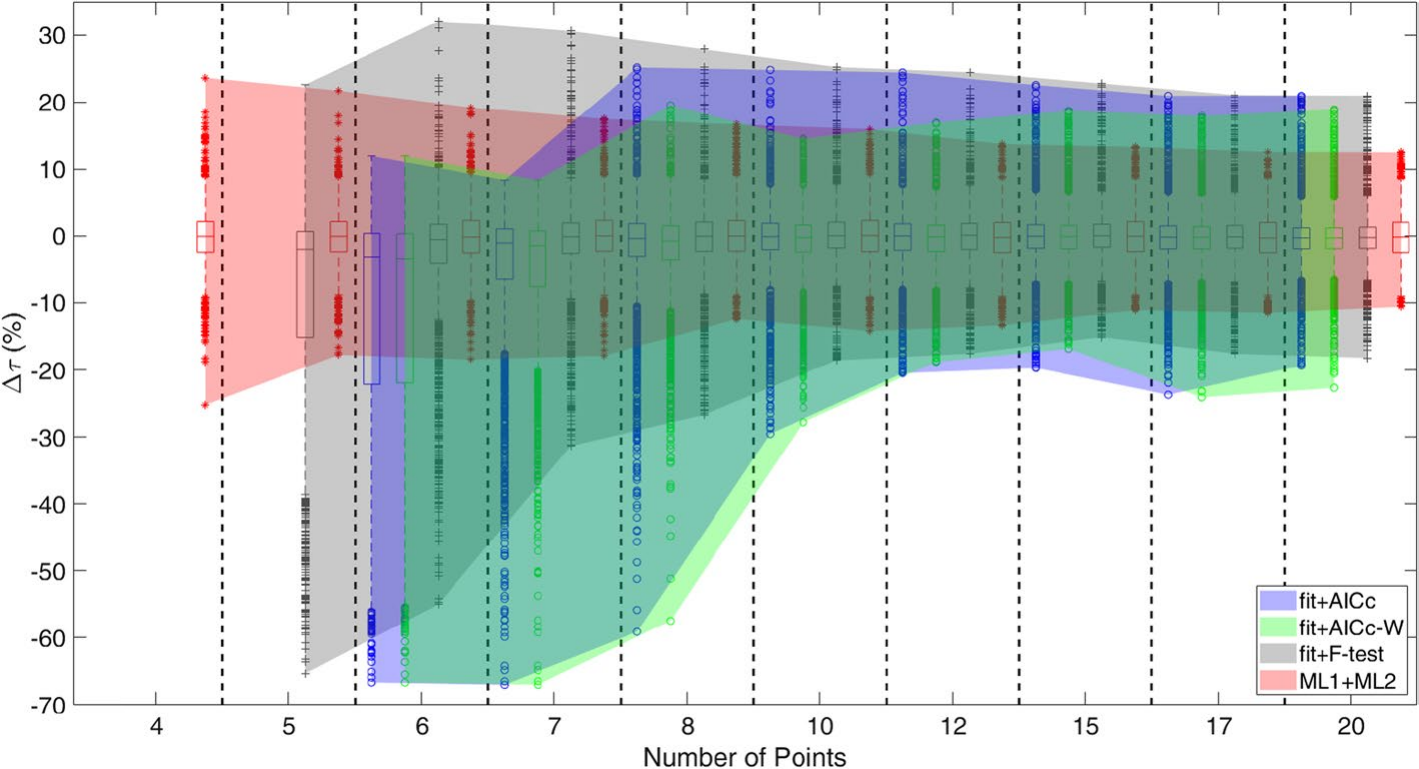
Boxplots representing the Δτ distributions and the MER bands obtained using the fit + AICc (blue), fit + AICc-W (green), fit + F-test (grey) and ML1 + ML2 (red). For the analytical methods, points were fitted with both models and the AICc and F-Test determine which AUC should be considered. For ML, first the best fit model was predicted and then the model parameters in order to calculate the AUC

The $\mathrm{\Delta \tau }$ distributions shown in Fig. 5 were obtained including any classification failure from AICc, AICc-W, F-test and ML1. Even in this case, varying N, the median values are approximately constant for all the considered methods, while interquartile range varies from 24.7 to 3.1% for the fit + AICc, from 24.6 to 3.1% for the fit + AICc-W and from 15.8% to 3.1% for the fit + F-test. The width remains constant around 4.5% for the ML1 + ML2 system. With the increase of the number of points, MER varies from [− 66.7; 11.9]% to [− 19.3; 20.9]% for fit + AICc, from [− 66.7; 12.0] to [− 20.7; 18.1] for fit + AICc-W, from [− 65.3; 32.1]% to [− 15.1; 20.9]% for fit + F-test and from [− 25.2; 23.6] to [− 10.6; 12.5]% for ML1 + ML2.

When N < 8, $\mathrm{\Delta \tau }$ distributions obtained by AICc and F-Test are asymmetrical, while for N > 8 the distribution width, obtained with the three methods, are equally distributed. The MER band of ML2 is always thinner than the other two.

**Fig. 6:**
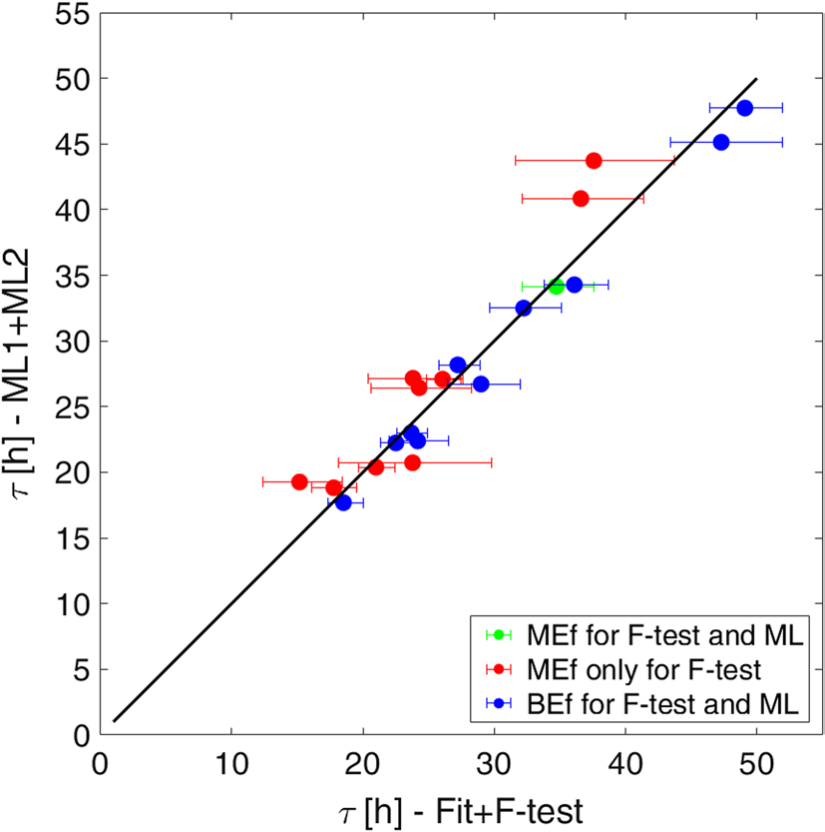
Comparison between the τ values obtained with ML1 + ML2 and Fit Algorithm + F-test. The red points represent the area under the curve of TACs classified as mono-exponential for the F-test while the blue points represent the area under the curve of TACs classified as bi-exponential for the F-test

Considering the TAC-Ps of 20 patients, 9 out of the 10 MEfs were classified as BEfs, while all the 10 presented BEfs were confirmed to be BEfs by ML1. Figure 6 shows the correlation between the $\tau$ calculated with ML1 + ML2 and the one calculated with the fit Algorithm + F-Test on the 20 patients data, obtaining an R^2^ equal to 0.93, and a difference percentage of the results in a [− 26.9;12.8]% range, with a median value of 0.2%.

## Discussion

The obtained results show the feasibility of using machine learning system both to classify the proper model and predict the TIAc with an improvement of the performances.

The training phase plays a crucial role into obtaining results close to true values, and one of the major advantages shown by ML is the possibility for the model to be trained with error-free data and then tested with data simulating real curves, resulting in a high CA (about 98.5%) and a $\mathrm{\Delta \tau }$ less than ± 25%. This aspect can be particularly advantageous in order to train the system without knowing the error of the measurement system “a priori”. Training with error-free data is not only advantageous but also recommended, as reported by Geron et al. [14]. The training phase should be conducted with the cleanest possible data, to avoid confusing the system and to prevent overfitting. To confirm this, training was performed with noisy data similar to the ones used in Tr_N_, and a decrease in classification accuracy of around 5% was recorded.

The time spent for training is very short, and it needs to be executed just once before running classifications and regressions.

The main difference between ML and the analytical method is the workflow. The former predicts the model and then assesses the biokinetic parameters. Instead, the latter calculates the biokinetic parameters for both models first and then chooses the optimal one. This aspect is crucial: the analytical method is not optimized because it necessarily performs both fit models and, in addition, the choice of the model is strongly dependent on the algorithm fit and on the goodness of the TIAc.

The cross-validation results are better than the ones obtained in the test phase, but this was to be expected: in fact, Ts_N_ simulates the measurements errors (thanks to the addition of Gaussian noise), and so the results get worse without being associated with an overfitting condition. Figure 3 shows that CA is not dependent on the number of points for ML1, while it is highly dependent for AICc and F-test. The analytical methods show a low accuracy when the number of points is near to the limit of applicability (from Eqs. 3 and 4 it is possible to notice that F-test and AICc are available only if the number of points is at least 5 and 6 respectively). As reported by Kletting et al. in [19], F-test and AICc classify as mono-exponential most of the bi-exponential curves (CA = 61.0% and CA = 50.0%) if the number of points is 5 or 6 respectively. In addition, the CA obtained with ML1, equal to 98.5%, is higher than the results obtained with AICc and F-test.

Figure 4 shows the best results obtainable through the two systems, dedicated to the assessment of biokinetic parameters. Considering an error-free classification, the width of the $\mathrm{\Delta \tau }$ distributions, obtained with ML2 and with the fit algorithm, are similar: hence, in most cases the two systems are equivalent. The MER obtained from ML2, represented by the red band in Fig. 4, does not exceed ± 15% and it is smaller than the one obtained using the fit algorithm (about ± 20% and represented by the blue band).

Glatting et al. in [4] use fitting algorithms that take into account uncertainties on individual points of the TAC. The fits used in the present study, on the other hand, do not consider such errors and, in future works, it will be necessary to perform a comparison. The use of these algorithms will likely lead to a convergence of performance between analytical methods and ML methods, but a priori knowledge of errors is not always possible. Therefore, “a priori”-trained ML systems remain a more general method for both model selection and obtaining kinetic parameters.

When the input to determine the biokinetic parameters are the classification results obtained through ML1, AICc, AICc-W and F-test, performances get worse for all the examined four systems (Fig. 5), but the ML1 + ML2 system keeps MER within ± 25%. The analytical methods tend to underestimate the area under the curve when the number of points is lower than 8. This effect is due to the tendency of the two methods to classify bi-exponential curves as mono-exponential.

The use of ML in this field can also lead to the possibility of using it in a more radical way. For example, an attempt was made to train the ML2 system to obtain the area under the curve directly as the output, instead of the fit parameters. The obtained results were similar to those shown in Figs. 4 and 5, but, according to the authors, this is an incorrect way of using ML systems: in fact they are already considered black boxes, and using the system in the aforementioned way, without the possibility to assure the goodness of the fit, can be challenging to justify in the clinical use of dosimetry.

It is important to underline that the performance of ML systems has proven to be fairly independent from the number of points composing the TACs, whereas a more pronounced dependence has been observed in relation to errors in the data. Some tests were conducted by varying the error on the points, resulting in a decrease in performance. These analyses were not reported in the results section because they go beyond the scope of this work, which is to demonstrate the feasibility of using ML systems in dosimetry in molecular radiotherapy, for model selection and the calculation of the area under the TAC curve.

**Fig. 7:**
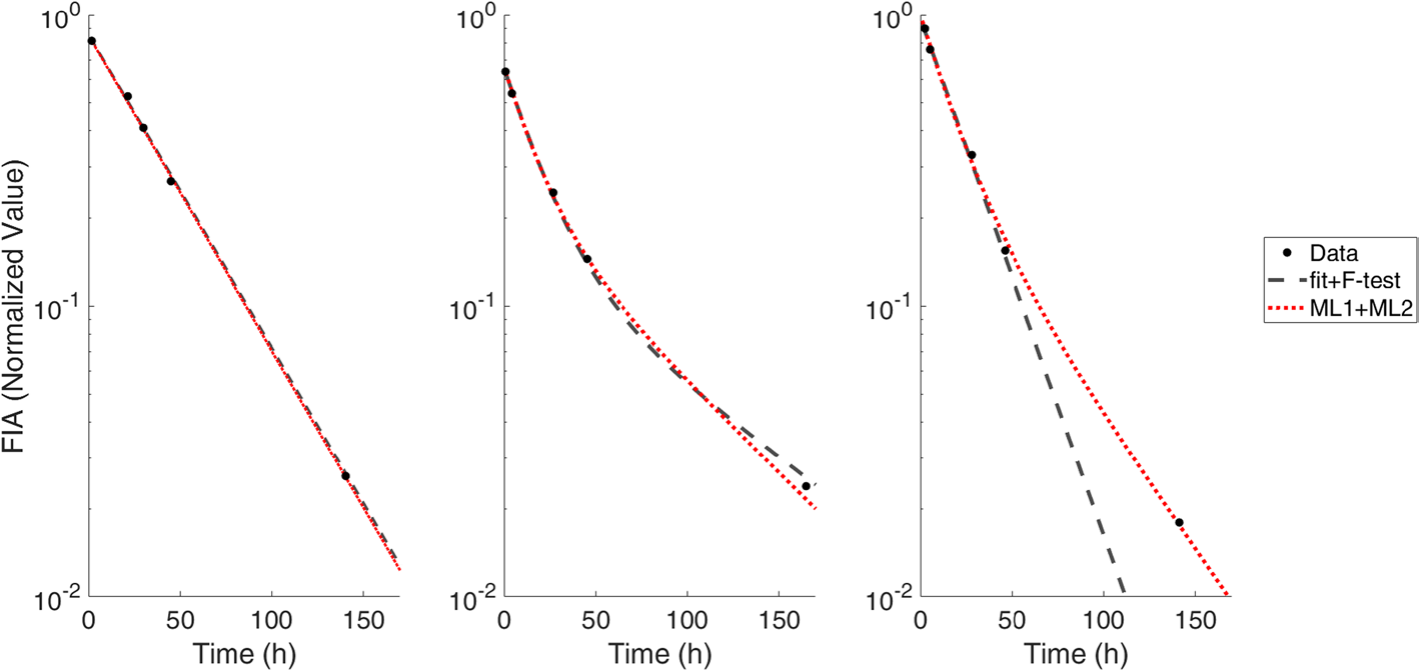
TACs of Wholebody FIA of three different patients and fit obtained with fit + F-test and ML1 + ML2 systems. **a** The two model agree classifying and fitting the data with a MEf. **b** Also in this case ML1 and F-test classify with a BEf. **c** In the third case the F-test classify the curve as MEf while it is a BEf for ML2. In this case the two obtained fit curves are significantly different

Figure 6 shows the applicability of the ML systems on real data: it confirms that, when ML1 and F-test classify the curve with the same model, the assessed biokinetic parameters are very similar. On the other hand, when classification does not coincide, the distance between the two results increases. An example of these results is shown in Fig. 7. Considering CA for 5 points (shown in Fig. 3) and the graph in Fig. 5, there is a high probability that the correct classification is given by ML1, and consequently the ML2 result could better represent the biokinetic curve of the patient.

ML1 and ML2 have been trained with curves simulating the whole-body FIA of patients administered with [^131^I]-NaI, but can be trained to perform cumulated activity calculations also for different radionuclides in metabolic radiotherapy, and with different dosimetry calculation methods (i.e. voxel dosimetry).

In 2017 Sarrut et al. [20] showed that the selection of the proper fit model reduces the number of fit failures in voxel dosimetry (defined as the case in which the optimizer does not converge and reaches the maximum number of iterations, or if the R^2^ is lower than a certain threshold). The brief time to perform the 2000 test fits and the independence of the performance from the number of points make these systems suitable for this application.

It is necessary to emphasize that each new application (such as the study of new curve models) needs a new training, and that the performance is strongly dependent on the similarity between the training samples and the clinical case. In addition, in this study, Gaussian-type errors were used on the data because the purpose of the work was to demonstrate the feasibility of using ML systems. However, in future studies, it will be necessary to investigate how data errors affect performance using different thresholds and various types of distributions, such as the Poisson distribution.

This is a study on the feasibility of using the ML system for classifying the correct fit model and predicting the TIAc, and it doesn’t have the purpose to study which ML algorithm is the optimal one in performing these two tasks. The choice of the logistic regression and the AdaBoost algorithm is arbitrary, and a subsequent study is necessary to identify which algorithms could increase the performance results.

In conclusion, to the knowledge of the authors, this study is the first to propose the use of ML systems for TIAc calculations for dosimetry in MRT. As demonstrated, the use is feasible and promising, but it requires further investigations in different fields. Therefore, investigations will be performed in order to train such systems with different algorithms, treatments, radionuclides, curve models and for voxel dosimetry.

## Data Availability

The data that support the findings of this study are available on request from the corresponding author.

## Appendix

### Method for synthetic TAC-Ps generation

The datasets (TR_N_ and TS_N_) used to train and test the systems consist of TAC-Ps, where each point composing them simulates the FIA_WB_ of patients with metastatic differentiated thyroid carcinoma (mDTC), administered with [^131^I]I-NaI. Each point in the TAC-Ps simulates the result of the geometric mean between measurements acquired from the anterior and posterior positions, using an external probe located 4 m from the patient. The TIAc values for the two bands are reported in Table 1 and are obtained using the upper and lower bounds of the TACs calculated for 50 patients administered in our institution and subjected to dosimetric study.

To generate each of the TAC-Ps, the following method was used for both mono-exponential and bi-exponential trends. For BEf, four time points were selected at 0, 24, 90, and 160 h and, for each of these times, a range of possible values was determined by the band showed in Fig. 2. From each of these ranges, a random FIA_WB_ value was extracted, resulting in 4 values corresponding to the 4 different times. These values were fitted with a bi-exponential curve, obtaining the parameters of the generated curve. Finally, a check is performed to ensure that the generated curve does not intersect the bands up to 500 h. If the check is successful, the curve parameters are recorded, and the TAC-Ps are created with the chosen number of points for either Tr_N_ or Ts_N_. The procedure for TAC-Ps with a mono-exponential trend is identical, with the only difference being that two points are extracted at times 0 and 90 h.
